# Supplementary material for: Integrating Metabolomics and Network Analyses to Explore Mechanisms of Geum japonicum var. chinense Against Pulmonary Fibrosis: Involvement of Arachidonic Acid Metabolic Pathway
Source: Int J Mol Sci. 2025 Feb 10;26(4):1462. doi: 10.3390/ijms26041462 (PMC11855089; doi:10.3390/ijms26041462)
Supplement: Supplementary file 1 [file ijms-26-01462-s001.zip › ijms-3403342-supplementary.pdf]

Contents

Figure **S1.** The structure of 26 compounds.

Figure S2. Multivariate statistical analysis results for the serum samples.

Figure S3. Effect of GJC aqueous extract on arachidonic acid metabolic pathway in PF mice.

Table S1. The relevant information for these 32 metabolites.

Table S2. "Compounds-Targets-Metabolites- Arachidonic acid metabolic pathway" network.

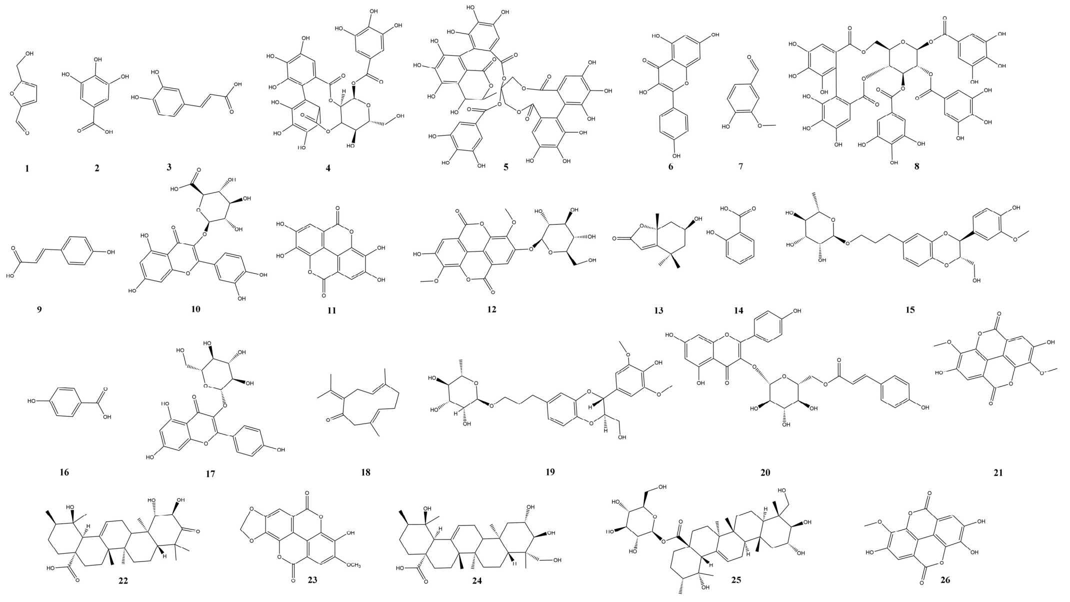

Figure S1. The structure of 26 compounds.

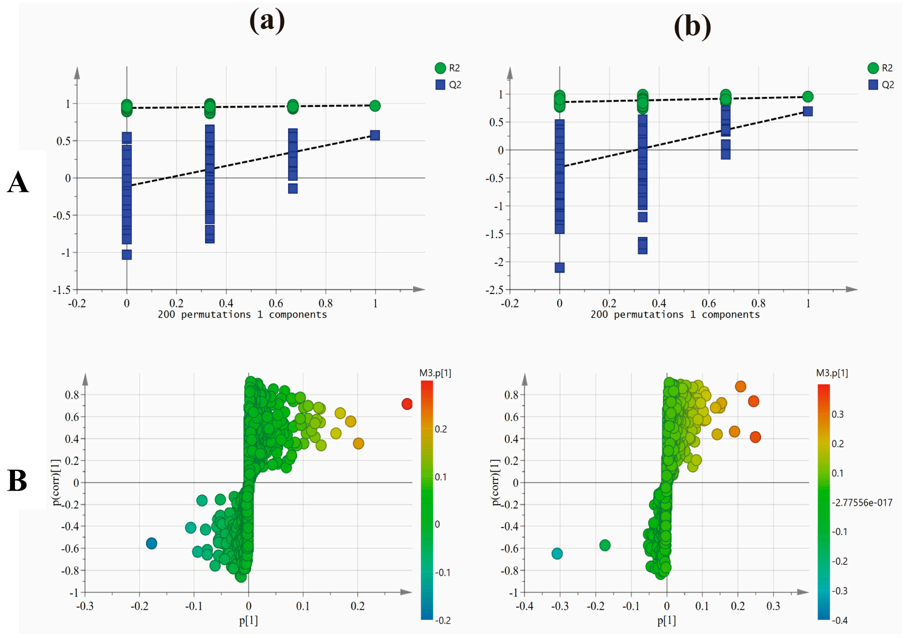

Figure S2. Multivariate statistical analysis results for the serum samples (n = 6). (a) negative ion mode and (b) positive ion mode. (**A**) Permutation test of the Control vs. BLM groups; (**B**) S-Plot score plot for OPLS-DA BLM of B.

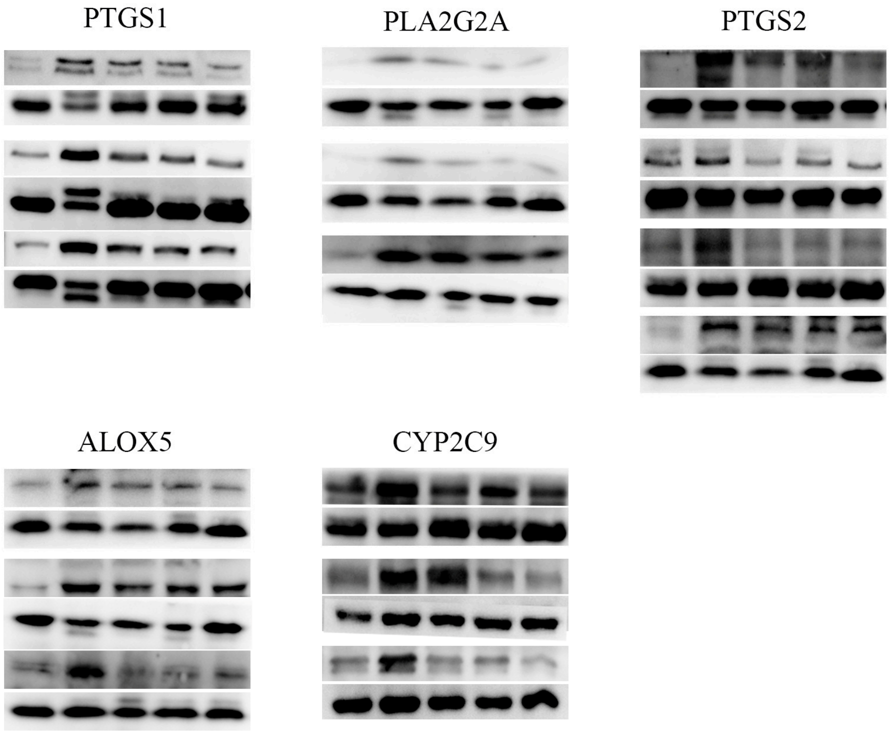

Figure S3. Effect of GJC aqueous extract on arachidonic acid metabolic pathway in PF mice.

Table S1. The relevant information for these 32 metabolites.

NO.	Metabolites	tR(min)	m/z	Class	Ion scan mode	KEGG	HMDB	VIP	P	Model vs Control	GJCH VS Model
M301T50	5-HETE	0.83	301.21721	Fatty Acyls	Negtive	C04805	HMDB0011134	4.69	0.0089	↑ **	↓
M303T50_2	Arachidonic acid	0.83	303.23289	Fatty Acyls	Negtive	C00219	HMDB0001043	15.01	0.0161	↑ *	↓ *
M227T50	Myristic acid	0.84	227.20131	Fatty Acyls	Negtive	C06424	HMDB0000806	2.94	0.0769	↑	↓
M137T59_2	2-aminobenzamide	0.98	137.071	Benzene and substituted derivatives	Postive	-	HMDB0033947	1.67	0.0026	↑ **	↓ *
M86T59	.gamma.-aminobutyric acid	0.99	86.06064	Carboxylic acids and derivatives	Postive	C00334	HMDB0000112	3.09	0.0025	↑ **	↓ *
M283T70	Octadecanoic acid	1.16	283.26408	Fatty Acyls	Negtive	C01530	HMDB0000827	6.68	0.0359	↑ *	↓ **
M177T102	Ala-Ser	1.70	177.06582	Carboxylic acids and derivatives	Postive	-	-	1.37	0.0152	↓ *	↑ *
M301T109	8-HETE	1.81	301.21717	Fatty Acyls	Negtive	C14776	HMDB0004679	1.40	0.0106	↑ *	↓
M92T216	Alanine-2,3,3,3-d4	3.59	92.04923	Carboxylic acids and derivatives	Negtive	-	-	1.09	0.0506	↓	↑
M300T236	Sphingosine	3.93	300.28948	Organonitrogen compounds	Postive	C00319	HMDB0000252	2.67	0.0075	↑ **	↓ *
M282T236	Psychosine	3.93	282.27892	Sphingolipids	Postive	C01747	HMDB0000648	2.20	0.0096	↑ **	↓ *
M117T276_1	2-hydroxy-2-methylbutyric acid	4.59	117.05453	Fatty Acyls	Negtive	-	HMDB0001987	5.14	0.0236	↑ *	↓ *
M508T321	1-(1z-octadecenyl)-sn-glycero-3-phosphocholine	5.35	508.37571	Glycerophospholipids	Postive	-	HMDB0013122	2.14	0.0403	↑ *	↓
M276T332	Gln-glu	5.54	276.13411	Carboxylic acids and derivatives	Postive	-	-	1.57	0.0005	↑ ***	↓
M102T363	N-nitroso-n-methyl-3-aminopropionic acid	6.05	102.05539	Organonitrogen compounds	Postive	-	-	1.02	0.0053	↓ **	↑
M207T364	DL-Phenylalanine	6.06	207.11273	Carboxylic acids and derivatives	Postive	C00079	HMDB0000159	5.00	0.0377	↑ *	↓

M173T375	L-Norleucine	6.25	173.12847	Carboxylic acids and derivatives	Postive	C01933	HMDB0001645	9.64	0.0004	↑***	↓
M167T409	6-(methylthio)purine	6.82	167.04841	Imidazopyrimidines	Postive	C16614	HMDB0060412	4.17	0.0006	↑***	↓
M132T410	D-alloisoleucine	6.84	132.10195	Carboxylic acids and derivatives	Postive	C21092	-	3.31	0.0373	↑*	↓
M118T412_2	Betaine	6.86	118.08645	Carboxylic acids and derivatives	Postive	C00719	HMDB0000043	15.48	0.0587	↓	↑
M153T418	2,6-dihydroxybenzoic acid	6.97	152.99493	Benzene and substituted derivatives	Negtive	C21298	HMDB0013676	1.59	0.0096	↑**	↓
M102T419_2	4-hydroxy-l-isoleucine	6.98	102.09176	Carboxylic acids and derivatives	Postive	-	-	2.17	0.0352	↑*	↓
M137T424	Siduron	7.07	137.07099	Benzene and substituted derivatives	Postive	C18435	-	4.20	0.0209	↑*	↓*
M157T432	Suberic acid	7.19	157.09715	Fatty Acyls	Postive	C08278	HMDB0000893	12.08	0.0068	↑**	↓**
M161T464	Pimelic acid	7.73	161.09204	Fatty Acyls	Postive	C02656	HMDB0000857	2.15	0.0121	↑*	↓
M153T466	2,4,7,9-tetramethyl-5-decyne-4,7-diol	7.77	153.11345	Organooxygen compounds	Postive	-	-	1.52	0.0279	↑*	↓
M173T492	N2-Acetylornithine	8.20	173.09237	Carboxylic acids and derivatives	Negtive	C00437	HMDB0003357	1.42	0.0397	↑*	↓*
M256T518	5-methylcytidine	8.63	256.09524	Pyrimidine nucleosides	Negtive	-	HMDB0000982	1.24	0.004	↑**	↓*
M214T528_2	Glycerylphosphorylethanolamine	8.80	214.04814	Glycerophospholipids	Negtive	C01233	HMDB0000114	4.22	0.0109	↑*	↓*
M232T598	Gly-Arg	9.96	232.12907	Carboxylic acids and derivatives	Postive	-	-	1.25	0.0037	↑**	↓*
M129T651	4-piperidinecarboxamide	10.84	129.10235	Piperidines	Postive	C00489	HMDB0000661	1.20	0.0007	↑***	↓
M170T664	Pyridoxine	11.07	170.09241	Pyridines and derivatives	Postive	C00314	HMDB0000239	1.09	0.0247	↓	↑*

Table S2. " Compounds-Targets-Metabolites- Arachidonic acid metabolic pathway" network

NO.	Name	Degree
-	PTGS2	23
-	ALOX5	18
-	PTGS1	14
-	Arachidonic acid metabolism	8
-	CYP2C9	7
-	5-HETE	5
-	Arachidonic acid	4
H3	Caffeic acid	4
H20	Tiliroside or cis-Tiliroside	3
-	PLA2G2A	3
-	8-HETE	3
H13	Loliolid	3
H9	tellimagrandin II	3
H12	3,3'-di-O-methylellagic acid-4'-O-β-D-glucopyranoside	3
H14	Salicylic acid	3
H26	3-O-Methylellagic acid	3
H1	5-Hydroxymethyl furfural	2
H2	Gallic acid	2
H15	Cupressoside A	2
H16	4-Hydroxybenzoic acid	2
H24	19α-Hydroxyasiatic acid	2
H25	Niga-ichigoside F1	2
H4	Sanguiin H-4	2
H6	Kaempferol	2
H10	Quercetin 3-O-β-D-Glucuronide	2
H11	Ellagic acid	2
H17	Astragalin	2
H18	(*7S*,*8S*)-5-methoxycupressoside A	2
H21	3,3'-di-O-methylellagic acid	2
H22	1, 2, 19-Trihydroxy-3-oxo-12-ursen-28-oic acid	1
H5	Casuarinin or Potentillin	1
H8	p-Coumaric acid	1
